# Integration and Vascular Ingrowth of a Collagen Meniscal Implant: A Case Report

**DOI:** 10.5704/MOJ.1907.007

**Published:** 2019-07

**Authors:** M Duarte-Silva, F Guerra-Pinto, N Camelo-Barbosa, P Beja-da-Costa

**Affiliations:** Department of Orthopaedics and Traumatology, Hospital de Cascais Dr. José de Almeida, Cascais, Portugal; * Department of Orthopaedics, Hospital Pedro Hispano, Matosinhos, Portugal; ** Department of Orthopaedics and Traumatology, Giga Saúde Clinic, Lisbon, Portugal

**Keywords:** meniscal implant, collagen scaffold, biological integration

## Abstract

Meniscectomy is the most common surgery in orthopaedics. The absence of meniscal tissue might be related to irreversible damage to the articular cartilage. Meniscal replacement is a tissue-engineering technique for post-meniscectomy syndrome. Its success depends on the implant integration which was vastly proven in animal model studies. Histological evidence is hard to obtain in humans due to ethical issues. We report a clinical case in which a collagen scaffold meniscal implant was harvested six months after implantation due to mechanical failure. Histological analysis was performed revealing vascularisation not only of the peripheral attachment of the implant but also on the anterior horn. These morphologic findings demonstrate that this implant allows the colonisation by precursor cells and vessels, leading to the formation of a fully functional tissue. This present report is one of the few independent reports of scaffold biological integration in the literature.

## Introduction

The meniscus has functions in load bearing and joint stability. Meniscectomy is associated with secondary cartilage damage^1^. Replacement of the meniscal tissue has led to the development of meniscal scaffold and allograft transplantation techniques. Successful implant integration is a central issue. There is evidence of integration in studies in animal models but, due to ethical issues, histological samples of integrated implants in humans are hard to obtain^2^. We report a clinical case in which a Collagen Meniscus Implant was harvested six months after implantation. The histological analysis of the fragment showed vascularisation throughout the whole implant. This report documents the first histological evidence of complete implant integration in a human subject from an independent group.

## Case Report

We report the case of a 28 years old female patient who underwent a right knee arthroscopy and partial meniscectomy at 26 years of age with short term relief of her mechanical symptoms. Seven months after the surgery she reported progressive medial knee pain, disabling and persistent, without meniscal symptoms. Her body mass index and limb alignment were normal and physical examination revealed no ligament laxity. The MRI showed post-surgical changes in the medial meniscus, without re-rupture, with intact anterior and posterior horns. The articular cartilage was reported as grade II/III disease in the medial compartment.

Our team performed a collagen scaffold meniscal transplantation, a porous collagen-glycosaminoglycan matrix composed of 97% purified type I collagen isolated and derived from bovine Achilles tendon. Surgery was done with an all-inside technique and the fixation of the collagen was achieved with Sequent Meniscal Repair Device® [ConMed^®^, NY, USA].

Rehabilitation consisted on restricted ROM (0 – 90 degrees) in the first four weeks and protected weight bearing for eight weeks. The patient completed her rehabilitation protocol at five months post-surgery, with normal gait and range of motion, with residual pain after walking for long periods.

Six months after allograft implantation, the patient suffered a new knee sprain with external foot rotation while going down a small flight of stairs. She complained of a new onset of medial knee pain and joint locking symptoms. MRI was performed immediately, but its results regarding scaffold integrity were inconclusive due to the high-intensity T1 and T2 signal changes.

The patient asked for an immediate surgical relief. Arthroscopic examination showed a fracture of the posterior third of the meniscal implant, with an unstable fragment of 10 x 6 mm. (Figure 1a, 1b) This fragment was yellow in color, similar to the remaining implanted meniscus, which was stable at inspection and hook probing. The stable implant represented 65% of the whole implanted tissue. The unstable scaffold fragment was removed and submitted for histopathological analysis, which revealed different processes occurring in the implant (Figure 2). The recovery of the patient was good, with a knee severity score of 88, three months post-operatively.

**Fig. 1a:**
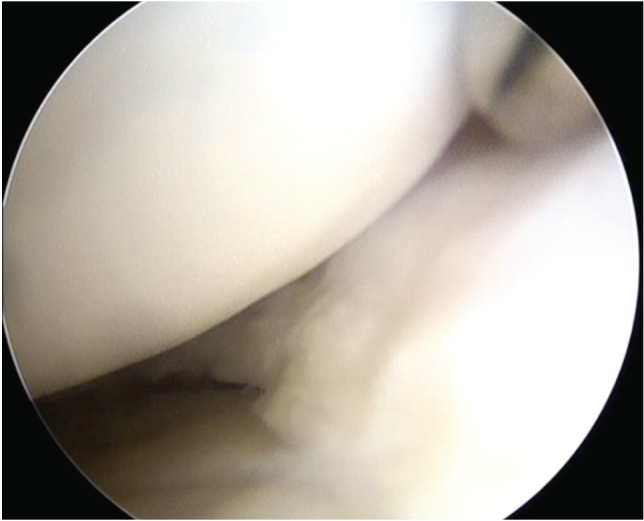
Arthroscopic image showing the integration of the meniscal implant in its body and anterior horn.

**Fig. 1b:**
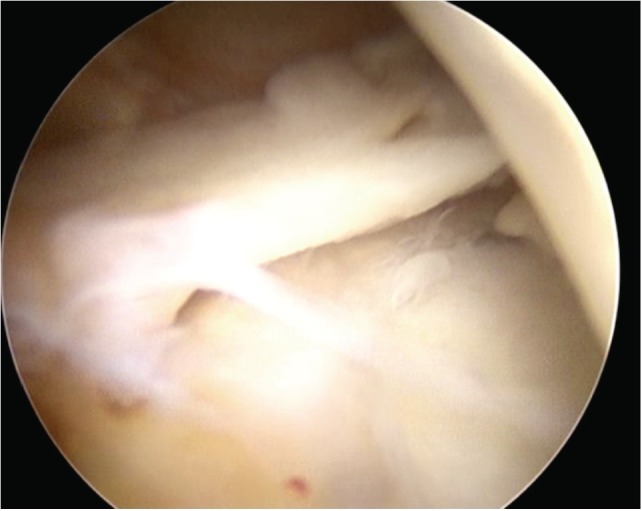
Arthroscopic image showing implant rupture in the posterior horn of the medial meniscus.

**Fig. 2:**
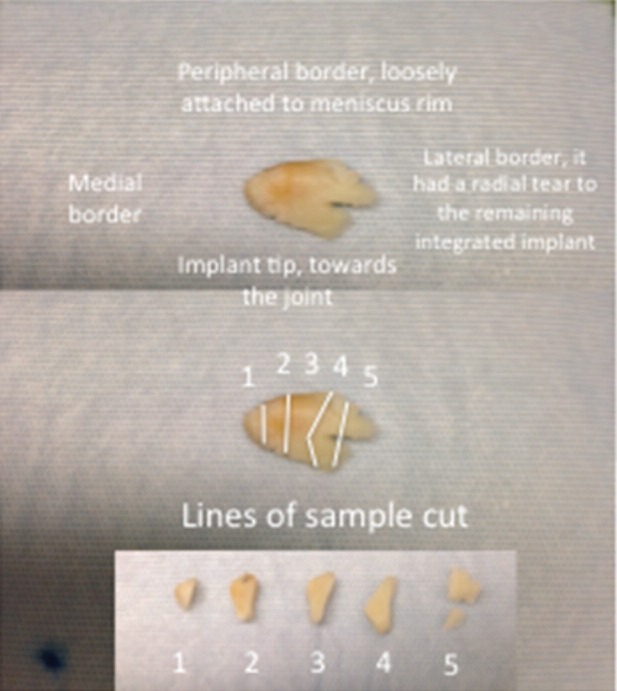
Excised fragment from the ruptured meniscal implant, and its lines of sample cut for histology analysis.

A reconstruction of the three microscopic photographs of the sagittal cut of the implant was done (Fig. 3a). A 40X amplification of the articular tip of the meniscus (Fig. 3b), and a 200X amplification of the periphery of the implant revealed distinctively identifiable vessels (Fig. 3c).

**Fig. 3a:**
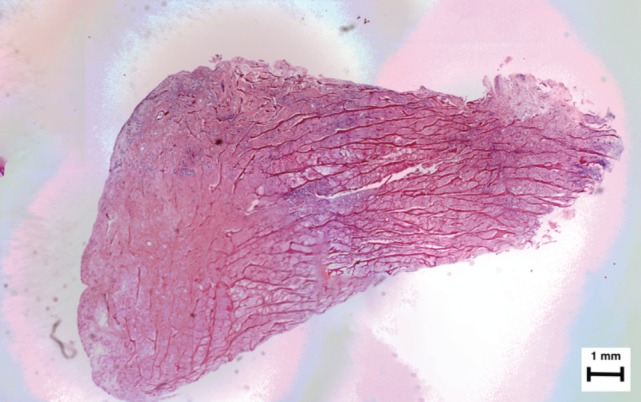
Hematoxilin-Eosin. Microscopic reconstruction. Please note the triangular shape, the presence of horizontal dark red lines and the filling with cells and matrix throughout the implant. The most important finding were the blood vessels in the periphery of the implant. The collagen fibers are present throughout the whole implant, but are less dense in the most peripheral area, attached to the meniscal rim.

**Fig. 3b:**
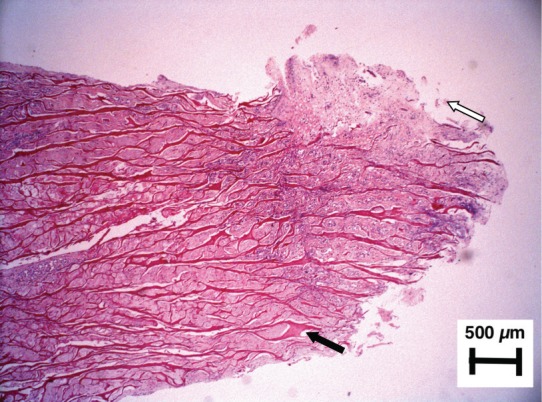
Hematoxilin-Eosin x40. Tip of the implant. Fibrochondrocytes filling the spaces between the collagen mesh (black arrow). Outer growth of fibrochondroid tissue covering and making the implant longer (white arrow) can also be observed.

**Fig. 3c:**
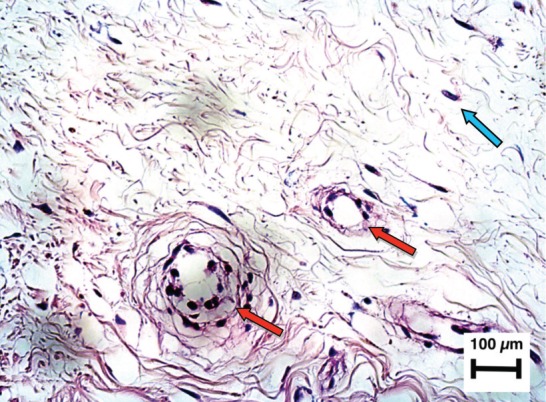
Hematoxilin-Eosin x 200. Blood vessels in the periphery of the implant. Not only were there fibro-chondrocytes filling the spaces between the collagen mesh, but also these cells escaped the mesh, covering it and making it longer. The vessels (red arrows) and the fibrochondrocytes (blue arrow) are seen.

## Discussion

The main goal of the implantation of meniscal collagen scaffold is to reduce pain, restore knee function and hopefully minimise long term joint disease after partial meniscectomy^2^.

The collagen scaffold is a product of animal origin and despite the risk of immunologic reaction^2^ no eosinophilia or synovitis was observed in our patient. The scaffold allowed cell migration and colonisation that enabled fibrochondroid meniscal-like tissue formation^3^.

Successful meniscal integration was demonstrated by Hansen *et al* by histological analysis of a collagen scaffold meniscus implant in canine model. Fibrochondroid tissue was observed at 1-year implantation in the model^3^.

Verdonk *et al* found the presence of matured collagen (type I collagen, fibroblasts and fusiform fibrochondroblast-like cells) in a second-look arthroscopy and biopsy 12 months after biodegradable polyurethane scaffold implantation in a human model^4^.

Histological biopsy analysis by Stone *et al* of a collagen scaffold on a second-look arthroscopy six months after implantation revealed presence of regenerated tissue similar to native fibrous meniscal cartilage^5^. In vitro studies have confirmed that porous collagen matrices can support cellular ingrowth and new matrix synthesis. Human studies have also shown that this implant appears to support regeneration even in the inner portions of the meniscus^5^.

These studies support the hypothesis that collagen meniscus implant possesses tissue-conductive properties for regeneration of meniscus-like tissue^2^.

Our findings are similar to Stone *et al*^5^, as we also found organised fibrochondroid tissue at six months after implantation, earlier than the Verdonk and Hansen studies^3,4^. This fact may be related to the different time points of implant resection or biopsy in their models.

Angiogenesis was observed from the periphery to the tip of the implant. A lesser degree of angiogenesis was observed in the tip of the implant comparing to its periphery but its presence outlined the potential of tissue in growth in the whole scaffold.

Although we cannot rule out biological failure as the cause for the implant rupture, the history of knee sprain suggests that mechanical trauma is the most likely cause for the tear. Our histological analysis demonstrated fibrochondroid cells ingrowth filling the spaces between the collagen mesh implant tissue at six months of implantation. Although the implant tissue was not fully replaced the time period for collagen scaffold degradation might be sufficient for meniscal tissue formation.
